# Cryo-EM structures of staphylococcal IsdB bound to human hemoglobin reveal the process of heme extraction

**DOI:** 10.1073/pnas.2116708119

**Published:** 2022-03-31

**Authors:** Omar De Bei, Marialaura Marchetti, Luca Ronda, Eleonora Gianquinto, Loretta Lazzarato, Dimitri Y. Chirgadze, Steven W. Hardwick, Lee R. Cooper, Francesca Spyrakis, Ben F. Luisi, Barbara Campanini, Stefano Bettati

**Affiliations:** ^a^Interdepartmental Center Biopharmanet-TEC, University of Parma, Parma 43124, Italy;; ^b^Department of Medicine and Surgery, University of Parma, Parma 43126, Italy;; ^c^Institute of Biophysics, National Research Council, Pisa 56124, Italy;; ^d^Department of Drug Science and Technology, University of Turin, Turin 10125, Italy;; ^e^Department of Biochemistry, University of Cambridge, Cambridge CB2 1GA, United Kingdom;; ^f^Department of Food and Drug, University of Parma, Parma 43124, Italy

**Keywords:** bacterial hemophores, IsdB, hemoglobin, cryo-EM

## Abstract

Iron surface determinant B (IsdB) is a hemoglobin (Hb) receptor essential for hemic iron acquisition by *Staphylococcus aureus*. Heme transfer to IsdB is possible from oxidized Hb (metHb), but inefficient from Hb either bound to oxygen (oxyHb) or bound to carbon monoxide (HbCO), and encompasses a sequence of structural events that are currently poorly understood. By single-particle cryo-electron microscopy, we determined the structure of two IsdB:Hb complexes, representing key species along the heme extraction pathway. The IsdB:HbCO structure, at 2.9-Å resolution, provides a snapshot of the preextraction complex. In this early stage of IsdB:Hb interaction, the hemophore binds to the β-subunits of the Hb tetramer, exploiting a folding-upon-binding mechanism that is likely triggered by a *cis/trans* isomerization of Pro173. Binding of IsdB to α-subunits occurs upon dissociation of the Hb tetramer into α/β dimers. The structure of the IsdB:metHb complex reveals the final step of the extraction process, where heme transfer to IsdB is completed. The stability of the complex, both before and after heme transfer from Hb to IsdB, is influenced by isomerization of Pro173. These results greatly enhance current understanding of structural and dynamic aspects of the heme extraction mechanism by IsdB and provide insight into the interactions that stabilize the complex before the heme transfer event. This information will support future efforts to identify inhibitors of heme acquisition by *S. aureus* by interfering with IsdB:Hb complex formation.

## Significance

During infection, the human pathogen *Staphylococcus aureus* expresses a surface-exposed receptor, Iron surface determinant B (IsdB), that captures free human hemoglobin (Hb) and removes heme to retrieve iron, an essential nutrient for bacterial proliferation inside the host. Using single-particle cryo-electron microscopy, we solved the structure of two complexes between Hb and IsdB that represent snapshots of the initial interaction, where heme is still bound to Hb, and the final complex after completion of heme extraction. The structural and dynamic details unlocked through these structures will boost the design of inhibitors of IsdB:Hb interaction that might work as innovative antimicrobials.

The interaction of pathogens with their hosts has been shaped by millions of years of coevolution, and among the adaptive mechanisms that have arisen is the highly effective acquisition of essential nutrients required for host colonization. In the case of *Staphylococcus aureus*, the essential element Fe can be obtained in hemic form from host hemoglobin (Hb), which is available in plasma and increases upon erythrocyte lysis caused by secreted bacterial hemolysin toxins (1). Heme extraction is mediated through the action of two receptors exposed on the surface of the bacterium: the Iron surface determinants (Isd) IsdB and IsdH (2, 3). IsdB and IsdH share high sequence and structural homology and a similar mechanism of action (4, 5); but IsdB is likely predominant during infection (2, 6, 7) and has been exploited for the development of vaccines (8). Interfering with IsdB activity might prove an efficacious approach to treat infections.

IsdB is a modular protein formed by three domains: two NEAT (NEAr iron Transporter) domains (IsdB^N1^ and IsdB^N2^) separated by an intervening linker domain (IsdB^L^) (9). NEAT domains present an immunoglobulin-like fold that binds heme and have been identified in many Gram^+^ bacteria, with higher prevalence in pathogens (10, 11). The two NEAT domains of IsdB play distinct roles in the process of heme extraction from Hb. IsdB^N1^ makes high-affinity binding to Hb through the surface-exposed loop 2 (11) and positions IsdB^N2^ for heme extraction. IsdB^N2^ carries the heme-binding motif (^440^YDGQY^444^) that is largely conserved in all NEAT domains of *S. aureus* (11). In the IsdB:heme complex Tyr440 in the motif coordinates the hemic iron and Tyr444 stabilizes the conformation of Tyr440 through H-bonding (12). Notably, IsdB can extract heme from the ferric form of Hb, metHb, and not from the ferrous forms, bound to either oxygen (oxyHb) or carbon monoxide (HbCO) (4, 13).

In its action, IsdB accelerates the rate of spontaneous heme release from Hb up to 2,000-fold (14). The rate-limiting step of the process has been identified, for the homologous hemophore IsdH, in the hydrolytic cleavage of the coordination bond between the proximal histidine and the heme iron (5), proposed to be assisted by the formation of a bis-histidine intermediate (4, 15, 16). The catalytic action of the complex is likely favored by conformational dynamics that have been difficult to capture by X-ray crystallography. Many approaches have been attempted to trap the IsdH/IsdB:Hb complex in a given step of the transfer process using extraction-deficient hemophore mutants and isolated NEAT domains (4, 17–21), and a structure has been determined of IsdB:Hb in which heme has been displaced to the receptor–Hb interface (4). Here we have used single-particle cryo-electron microscopy (cryo-EM) to determine the structure of two complexes of IsdB with human Hb. The first complex, IsdB:HbCO, is unable to complete heme extraction from Hb and transfer to IsdB, but reveals at 2.9 Å resolution the interprotein interactions preceding heme extraction; the second complex, IsdB:metHb, depicts the posttransfer complex. While the latter complex has a limited resolution of 5.8 Å, it shows the final step of heme acquisition by IsdB.

## Results

### Identification of Conditions to Trap Stable and Homogenous Complexes of IsdB with Hb.

**Fig. 1.**
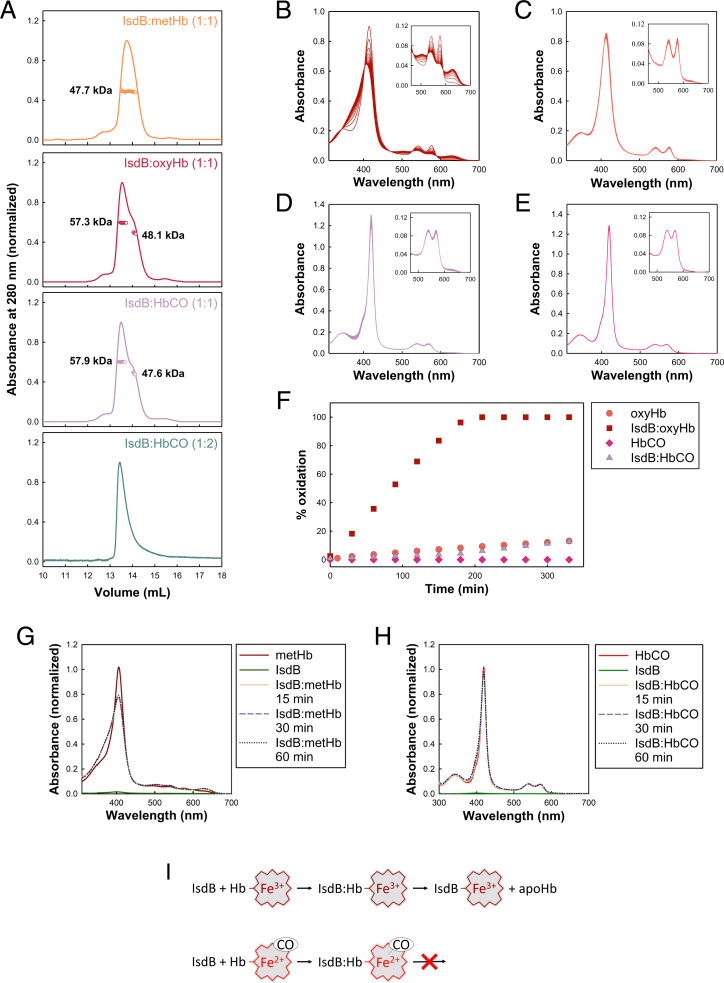
Biochemical characterization of IsdB interaction with metHb, oxyHb, and HbCO. (*A*) SEC-MALS analysis of different IsdB:Hb complexes samples at 1 g/L concentration. Absorbance (lines) and weight average of MW (dots) are plotted versus the elution volume, showing constant molar mass values over the entire peak width. The stoichiometric ratio used to prepare the samples is indicated in parentheses for each run (1:1, one IsdB to one globin chain; 1:2, one IsdB to one globin dimer). (*B*–*E*) Time-resolved spectra of either oxyHb in the presence (*B*) and absence (*C*) of IsdB or HbCO in the presence (*D*) and absence (*E*) of IsdB. (*F*) Time course of Hb autoxidation calculated by spectral deconvolution of data presented in *B* to *E*. (*G* and *H*) Absorption spectra of metHb (*G*) and HbCO (*H*) before and after the addition of IsdB. The Soret peak of metHb at 406 nm decreases and blue shifts and the absorption at 380 nm increases after addition of IsdB, while no spectral changes are observed when IsdB is mixed with HbCO. Both signals were stable for at least 1 h at 4 °C, validating the stability of the complexes in the time frame required for preparation of cryo-EM specimens. In the IsdB:metHb complex heme is bound to IsdB, while in the IsdB:HbCO complex the cofactor is neither transferred to the hemophore nor oxidized. (*I*) Schematic of IsdB:Hb interaction and heme extraction. Although IsdB binds both metHb and HbCO, it can only extract heme containing oxidized iron.

We sought to trap the IsdB:Hb complex in two states, before and after heme extraction, that had not been isolated previously. To estimate the molecular mass of the IsdB:Hb complexes in these states and thus their oligomerization status, we initially performed size-exclusion chromatography and multiangle light scattering (SEC-MALS) analyses (Fig. 1*A* and *SI Appendix*, Fig. S1). The calculated molecular weight (MW) for IsdB was 42.3 kDa, which is close to the expected value of 43.3 kDa. We observed that chemically cross-linked oxyHb has an apparent MW of 62.3 kDa (*SI Appendix*, Fig. S1*B*), as expected for the tetrameric protein (calculated MW = 64 kDa). However, metHb, oxyHb, and HbCO eluted as single peaks with MWs of 38.5, 47.8, and 46.2 kDa, respectively (*SI Appendix*, Fig. S1*A*), that are smaller than the expected mass of the tetramer, but consistent with an ensemble average from a dimer–tetramer equilibrium (32 to 64 kDa). The ensemble behavior is corroborated by the observed concentration-dependent increase of the calculated MW of oxyHb from 43.8 to 55.2 kDa (*SI Appendix*, Fig. S1*B*). The trends in the experimental mass averages of metHb, oxyHb, and HbCO are in agreement with our earlier findings of a higher K_D_ for tetramer dissociation of oxidized Hb compared to the oxyHb and HbCO forms (13).

Keeping in mind that the SEC profiles correspond to stable mixtures of species and MALS provides average ensemble masses, we used SEC-MALS to identify the conditions under which relevant assemblies could be isolated for further structural analysis. Initially, we chose a stoichiometric ratio of 1:1 (one IsdB to one globin chain) for complex formation of IsdB:metHb, IsdB:oxyHb, and IsdB:HbCO, based on previous studies on metHb (13). The IsdB:metHb complex elutes in a single peak (Fig. 1*A*) and the elution profile at 280 nm closely matches the chromatogram collected at 406 nm where the heme absorbs maximally (*SI Appendix*, Fig. S1*E*). However, the shape and the imperfect overlap of the chromatograms collected at the two wavelengths suggest the peak is likely a stable mixture of species with an average MW of 47.7 kDa. The dominant species appears to be a complex formed by one IsdB bound to a single Hb chain (59 kDa) and the sample does not contain significant amounts of free Hb, free IsdB, or higher-order complexes (2IsdB:Hb_dimer_ complex [118 kDa] or 4IsdB:Hb_tetramer_ complex [236 kDa]). As Hb monomerization usually occurs at extremely low concentration (K_D_ in the picomolar range) (22), this result suggested that heme extraction upon IsdB binding leads to Hb dimer destabilization (23). When IsdB is combined with either oxyHb or HbCO in 1:1 stoichiometric ratio, two partially resolved peaks are visible in the chromatogram, corresponding to masses of 57.3/57.9 and 48.1/47.6 kDa (Fig. 1*A*). Importantly, only the first peak significantly absorbs light at 415/419 nm and therefore contains heme (*SI Appendix*, Fig. S1 *F* and *G*). When the stoichiometric ratio was changed to 1:2 (one IsdB to one globin dimer), a single sharp peak was detected, indicating that the low-molecular-weight species previously observed likely corresponded to free IsdB (Fig. 1 *A*, *Bottom*).

The preparation of a stable complex between IsdB and Hb that represents the preextraction state requires a liganded Hb that does not autoxidize in the time frame of cryo-EM specimen preparation. Both oxyHb and HbCO undergo autoxidation, and it has been speculated that IsdB accelerates this process to account for the efficiency of heme transfer. We measured the fraction of oxidized Hb at 37 °C and pH 7.4 in the presence and absence of IsdB by deconvolution of absorption spectra (Fig. 1 *B*–*F*). As expected, the rate of autoxidation is significantly slower for HbCO than for oxyHb. The autoxidation process accelerates significantly in the presence of IsdB, which causes the complete oxidation of oxyHb in less than 4 h. Again, the HbCO form is more stable and the fraction of oxidized Hb in complex with IsdB is only 0.13 after 5 h. This finding indicates that IsdB:HbCO is stable enough to prevent heme transfer during specimen preparation and thus to allow trapping of the initial preextraction complex. We further confirmed that heme is not transferred from HbCO to IsdB by monitoring the transfer reaction by absorption spectroscopy under conditions similar to the grid preparation process (Fig. 1 *G* and *H*). Indeed, the complexes between IsdB and Hb carrying heme in the oxidized or the reduced ligated form present distinctive absorption peaks related to the environment experienced by heme and to the iron oxidation state. The Soret peak represents the main signature of the heme state, and the formation of the complex between IsdB and metHb is associated with the rapid transfer of the cofactor to the hemophore, identified by a change in the absorption at 406 and 380 nm (4, 6, 13, 24). Conversely, the Soret peak remains unaffected after IsdB:HbCO complex formation, indicating that the reduced heme is stably bound to Hb (Fig. 1 *G–I*). For cryo-EM analysis we prepared the grids of the preextraction complex using a 1:2 IsdB:HbCO stoichiometric ratio, while we used a 1:1 IsdB:metHb stoichiometric ratio for the preparation of the final complex with heme transferred to IsdB.

### IsdB Complex with HbCO: A Snapshot of the Step Preceding Heme Extraction.

**Fig. 2.**
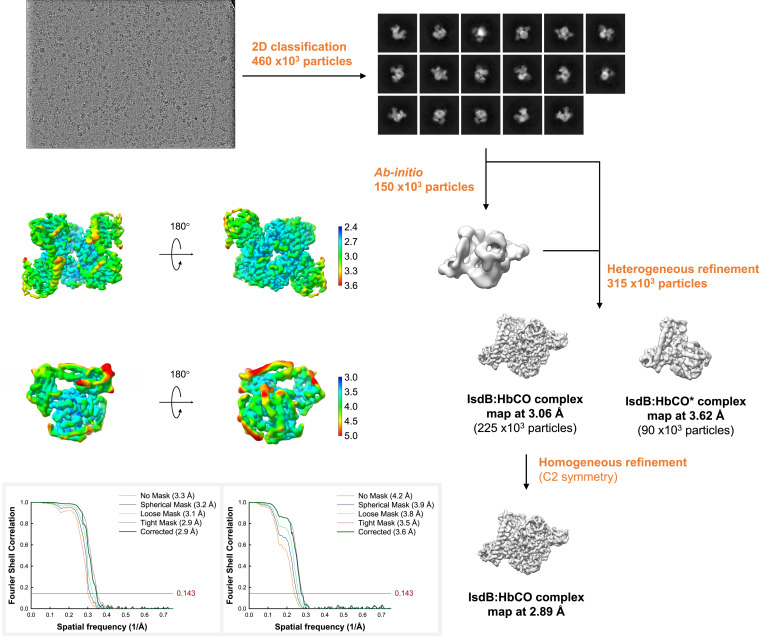
Single-particle analysis of IsdB:HbCO and IsdB:HbCO* complexes. *Top* shows a representative micrograph and selected 2D class averages used to generate initial reference maps and refined maps (*Right* flowchart). *Middle Left* shows the local resolution cryo-EM density maps in two orientations for the IsdB:HbCO (1:2) and IsdB:HbCO* (1:1) complexes. *Bottom Left* shows the Fourier shell correlations for the two models with different solvent masks and the estimated resolution of the optimal reconstructions.

**Fig. 3.**
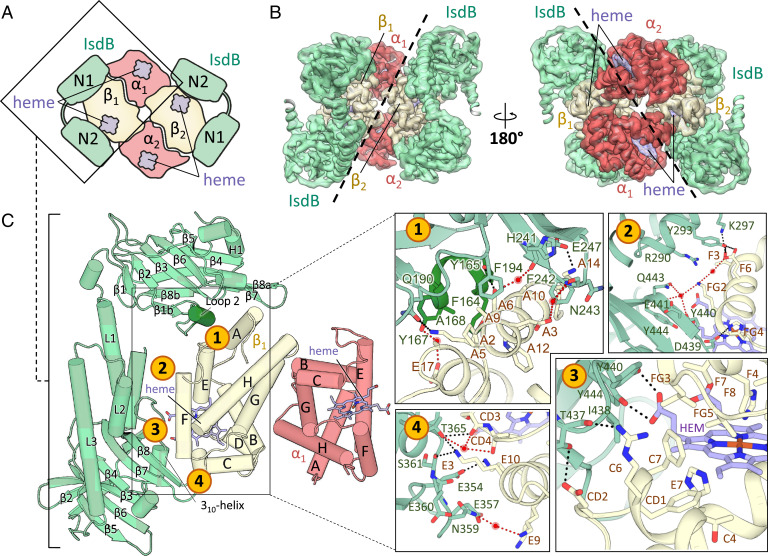
IsdB:HbCO complex structure from cryo-EM. (*A*) Schematic representation of IsdB:HbCO complex. (*B*) Top and bottom views of the 2.9 Å cryo-EM map and the refined model. (*C*, *Left*) Asymmetric unit of the IsdB:HbCO complex, containing one IsdB and an αβ-Hb dimer. Secondary structure elements are labeled and loop 2 is highlighted. (*C*, *Right*) Zoomed-in view of the principal interacting areas of IsdB:HbCO indicated on the *Left*: zone 1, molecular contacts between Hb and IsdB^N1^ that are expected to promote complex formation; zone 2, contacts of IsdB^L^ and IsdB^N2^ with F helix; zones 3 and 4, network of interactions between IsdB^N2^ and the heme-binding pocket of Hb.

**Fig. 4.**
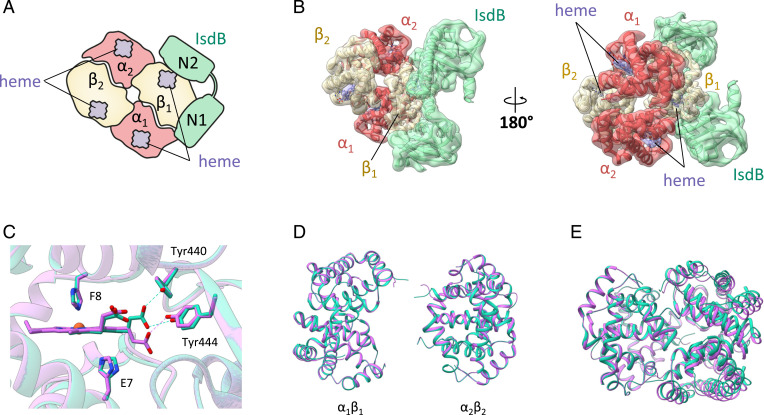
IsdB:HbCO* complex structure from cryo-EM. (*A*) Schematic representation of IsdB:HbCO* complex. (*B*) Top and bottom views of the 3.6 Å cryo-EM map and the refined model. (*C*) Heme-binding pocket on β-Hb chains of IsdB:HbCO (cyan) and IsdB:HbCO* (purple) complexes. Key residues are shown as sticks and polar contacts between IsdB residues and heme are depicted with dashed lines. (*D*) Alignment of separated α_1_β_1_ and α_2_β_2_ Hb dimers in IsdB:HbCO (cyan) and IsdB:HbCO* (purple) complexes. (*E*) Superposition of Hb tetramers in the IsdB:HbCO complex (cyan) and in the IsdB:HbCO* complex (purple).

The three-dimensional (3D) reconstruction of two major compositional states (Fig. 2 and *SI Appendix*, Table S1) was obtained at resolutions of 2.9 and 3.6 Å, respectively, as determined by the Fourier cell correlation (FSC )= 0.143 criterion (Fig. 2). The dominant state (IsdB:HbCO, more than 70% of the particles; Protein Data Bank [PDB] ID 7PCH) is the complex comprising two IsdB molecules binding the β-Hb subunits of a Hb tetramer (Fig. 3). The less populated state (IsdB:HbCO*, PDB ID 7PCQ) corresponds to one IsdB molecule bound to a β-Hb subunit of a Hb tetramer (Fig. 4).

The 1:2 IsdB:HbCO model has the highest resolution obtained thus far for a complex between a wild-type *S. aureus* hemophore and human Hb. IsdB folds with its characteristic dumbbell structure, placing the IsdB^N1^ domain next to A and E helices of Hb and the heme-extracting IsdB^N2^ domain at the portal of the heme-binding pocket (Fig. 3 *A* and *B*). Despite this arrangement, heme remains bound to the Hb subunits. IsdB^N2^ also approaches the α-Hb chain of the opposite αβ-Hb dimer, forming a minor interface with area 106 Å^2^. The main interface between the β-Hb chain and the hemophore measures 1,200 Å^2^. IsdB also interacts with the heme prosthetic group exposed at the β-Hb subunit (62 Å^2^). Notably, the resolution of the cryo-EM map allowed the manual placement of water molecules, some of which mediate protein–protein contacts (Fig. 3*C*).

A detailed analysis of the complex reveals four interaction hotspots between IsdB and β-Hb chains, shown in Fig. 3*C* and summarized in *SI Appendix*, Tables S2 and S3. Some of the key polar interactions identified are similar to those seen in the related IsdH receptor and in agreement with the 3.6 Å resolution structure of the complex between metHb and a proteolytic fragment of IsdB that lacks the NEAT2 domain (4). One interaction network (zone 1, Fig. 3*C*) comprises Hb residues belonging to A and H helices and the EF loop that interact directly or through bridging water with residues Phe164-Ala168 (loop 2), Gln190, Phe194, Phe242, and Asn243 on IsdB. Notably, the loop 2 contains the Hb-binding motif (3, 18) and folds in a stable α-helix as also observed in the complex of Hb with isolated IsdB^N1^ and IsdH^N1^ domains (4, 9, 11, 18). The folding upon binding of loop 2 is likely associated to a prolyl *cis/trans* isomerization (vide infra) and represents a driving force in stable complex formation. Once folded, the helix intercalates in a hydrophobic groove lined by residues Val11 (A8), Trp15 (A12), and Leu75 (E19), between helices A and E of Hb. The second area (zone 2, Fig. 3*C*) corresponds to the interaction of IsdB^L^ and IsdB^N2^ with the F helix of Hb. The IsdB^L^ establishes a network of direct or water-mediated polar contacts with the F helix, involving Thr87 (F3) and Glu90 (F6) on Hb and Tyr293 and Lys297 on IsdB (*SI Appendix*, Table S2). These contacts likely contribute to F helix distortion, a crucial step for heme extraction, as demonstrated by the observation that isolated IsdB^N1^ and IsdB^N2^ domains simultaneously binding to Hb are not competent for heme extraction (25), but the F helix in the β-Hb subunits within the IsdB:HbCO complex appears unaltered with respect to native HbCO. Recent work by Andersen and coworkers (21) reports the structure of the complex between IsdH and a haptoglobin-bound Hb. Hb, in the oxidized, dimeric form is bound to two IsdH molecules on both α- and β-subunits, but the presence of haptoglobin prevents heme transfer. Interestingly, the interactions of α- and β-subunits of Hb with IsdH are not equivalent, and the Glu90 (F6)-Tyr495 (equivalent to Tyr293 on IsdB, belonging to the IsdB^L^) hydrogen bond is present only on the β-subunits. Notably, none of the interactions of IsdB^L^ with Hb observed in our structure were reported in the IsdB:Hb complex where IsdB binds to α-subunits (4), likely because the partial unfolding of the F helix hampered the correct positioning of residues. Hence, any involvement of the IsdB^L^ in driving selectivity for the binding of IsdB to β-subunits requires further investigation. Asp439 on the β7-β8 turn, which belongs to the IsdB^N2^ domain, interacts with His97 (FG4) (vide infra, Fig. 3*C*).

The third and fourth contact areas (zones 3 and 4, Fig. 3*C*) include interactions mediated by the IsdB^N2^ domain. Within zone 3, the hemophore interacts with the entrance of the heme-binding pocket in the β-Hb chain. Importantly, our structural analysis did not require stabilizing mutations and provides details of the preextraction interactions of a hemophore with the heme and its binding pocket in a wild-type protein. Hb residues interacting with IsdB are on the E helix and the intrahelical CD loop, while in IsdB residues are located mainly on the β7-β8 turn and on the β8 strand. Here, Tyr440 and Tyr444 directly contact the same heme propionate, whereas Asp439 on the β7-β8 turn interacts with His97 (FG4). These residues together with the 3_10_-helix (residues ^362^MMDTF^366^) and the β7-strand have been shown to form a handclasp complex that assists heme transfer in other proteins of the Isd system (26). Importantly, zone 3 shows van der Waals interactions that can favor heme extraction, as IsdB residues Ile438 and Tyr440 contact Phe41 (C7), Leu91 (F7), and Leu96 (FG3) in the β-Hb chain, which are part of the heme pocket. An analogous hotspot has been proved essential in IsdH and mutations at this level resulted in a higher activation energy barrier for heme transfer and a lower heme extraction rate (15). In Fig. 3*C*, a close-up of the interactions mediated by the 3_10_-helix and the loop region spanning residues 354 to 361 on IsdB^N2^ is shown (zone 4). This hotspot engages critical residues on the CD loop and E helix of the β-Hb chain, which are mainly involved in heme stabilization. In fact, the CD loop is necessary for distal histidine stabilization (27), with Lys59 (E3), Lys65 (E9), and Lys66 (E10) forming electrostatic interactions that stabilize the heme group (16). Overall, this structure depicts a complex where some critical interactions have been established that precede heme removal from Hb. Tyr440 and Tyr444 directly bind the cofactor propionates and the IsdB^L^ forms multiple contacts with the F helix. In the free, holo IsdB^N2^ Tyr440 directly coordinates the heme iron and Tyr444 H-bonds Tyr440, likely stabilizing its position (12). The role of Tyr440 and Tyr444 in participating in the extraction process has been largely supported, given their conservation in all NEAT domains of the Isd system (11, 17) and the deficiency in heme extraction of Y440A and Y444A mutants (4, 13).

Notably, density is absent for the CO molecule bound to the four prosthetic groups, but the heme retains the planar configuration typically found when a ligand occupies the axial position (*SI Appendix*, Fig. S2). The visible absorbance spectrum of HbCO mixed with IsdB is stable for at least 1 h under the conditions used for specimen preparation, suggesting that in solution complex formation with the hemophore is not causing the ligand loss (Fig. 1*H*). Compared to the HbCO crystal structure, the distal histidine has moved toward the inside of the heme-binding pocket in the IsdB:HbCO complex to occupy some of the space that would otherwise accommodate the CO molecule (*SI Appendix*, Fig. S2*B*). The apparent CO loss might be due to disorder of the ligand or to oxidation of the heme or photolysis triggered by inelastically scattered electrons (28, 29). The released CO would likely be delocalized within a nearby internal binding pocket and, at 2.9 Å resolution, would be unresolved. At cryogenic temperatures, the Hb conformation will most likely remain unchanged (30).

The near-atomic resolution of the IsdB:HbCO complex density map allowed us to clearly identify Lys172-Pro173 and His369-Pro370 peptide bonds in the *cis* configuration. Only 5% of X-Pro peptide bonds are in this configuration (31) but peptidyl-prolyl *cis*/*trans* isomerization can act as a regulatory molecular switch in protein–protein interactions (32–34). His369 and Pro370 are near the 3_10_-helix that forms the handclasp complex involved in heme transfer (26). In IsdB the 3_10_-helix is formed by residues 362 to 366, and His369 directly contacts Asp364 and Val367. This X-Pro peptide bond in *cis* configuration is conserved in all the NEAT domains of the Isd system in charge of heme binding and transfer (PDB IDs 2E7D, 2ITE, 2ITF, 2O6P, 3VUA, 4XS0, 6TB2) (10, 20, 21, 35, 36) irrespective of their heme-binding state (either apo or holo); we thus conclude that isomerization of this bond is not associated with complex formation. On the other hand, Lys172 establishes polar contacts with residues Tyr167 and Ser170 that are in the Hb-binding motif (3) that folds in a α-helix when bound to Hb. Notably, in the solution structure of free IsdB^N1^ (PDB ID 2MOQ) (9) the Lys172-Pro173 bond is in the *trans* configuration, and the Hb-binding motif is unstructured (*SI Appendix*, Fig. S3*A*), suggesting that *cis*/*trans* isomerization of this peptide bond might represent a molecular switch for productive binding and assist loop 2 folding upon binding. The role of Pro173 in complex formation and heme extraction was assessed using the P173A variant (IsdB^P173A^). The dissociation constant of IsdB^P173A^ for Hb is at least one order of magnitude higher than that measured with the wild-type IsdB by an enzyme-linked immunosorbent assay (ELISA) (*SI Appendix*, Fig. S3*B*), which is in the nanomolar range, and suggests that isomerization of Pro173 to the *cis* configuration significantly contributes to binding affinity. This decrease in binding affinity does not hamper heme extraction from Hb under the experimental conditions of our assay (*SI Appendix*, Fig. S3*C*), which requires high protein concentrations (low micromolar range) to obtain a measurable absorbance signal from heme. High protein concentrations likely stabilize the weaker complex between Hb and IsdB^P173A^ and allow for heme transfer. However, while the complex of IsdB with metHb remains stable after heme extraction and no free Hb is visible in the SEC-MALS chromatogram (Fig. 1*A*), the Pro > Ala substitution destabilizes the complex and favors its dissociation after heme extraction (*SI Appendix*, Fig. S3*D*). Sodium dodecyl sulfate polyacrylamide gel electrophoresis (SDS-PAGE) analysis on eluted peaks confirms that they correspond to free IsdB and free Hb (*SI Appendix*, Fig. S3*E*).

For the less populated state IsdB:HbCO* (Fig. 4), the interface area between the hemophore and the β-Hb chain is comparable to the IsdB:HbCO state (1,200 and 1,209 Å^2^), and most of the contacts are conserved. However, the interaction between IsdB-Tyr440 and heme, which might have a critical role in the extraction of the cofactor from Hb, is not present (Fig. 4*C*). Small adjustments at the level of several loop regions were observed in IsdB; however, they are not involved in the interaction with Hb. By contrast, larger differences are observed for the structure of Hb in the two complexes, with a significant structural rearrangement of the C-terminal loops of both α-Hb chains. Moreover, even if the separate Hb dimers are highly superposable, with a rmsd of 0.162 and 0.219 Å (Fig. 4*D*), their relative position varies within the two complexes, thus resulting in a different quaternary organization (Fig. 4*E*). This less populated species could represent the first or principal binding event of the membrane-anchored IsdB, potentially on route to forming the complex hereby referred to as IsdB:HbCO.

Earlier studies of Hb in complex with hemophores indicated a quaternary arrangement that more closely resembles the T state (4, 20), but in both cases Hb has oxidized during the crystallization process and this might have influenced the quaternary organization. We have compared the quaternary structure of HbCO within the two complexes obtained in the cryo-EM analysis to the quaternary structures of T-state Hb (PDB ID 2DN2), some reference liganded forms (PDB IDs 2DN1 [Hb R-state], 1BBB [Hb R2-state], and 1YZI [Hb R3-state]), and Hb bound to IsdH^N2N3^ (PDB ID 4XSO) or IsdB (PDB ID 5VMM). We exploited the BGH frame (a region of invariant structure within the αβ dimer) (37, 38) on α_1_β_1_ dimers to manually superimpose Hb tetramers and used the DynDom software (39) to calculate interdimer translations and rotations. In Table 1, the rmsd is reported for α_1_β_1_ and α_2_β_2_ dimers, and rotations/translations are evaluated for the α_1_β_1_ dimer with respect to the α_2_β_2_ dimer. Our analysis indicates that the quaternary structure of the Hb tetramer is definitely more similar to that of liganded Hb, with the IsdB:HbCO complex showing the smaller rmsd and interdimer rotation/translation, when compared to R, and the IsdB:HbCO* complex with respect to the R2 state. This result corroborates that our cryo-EM models describe the IsdB complex with a Hb whose quaternary arrangement, in the absence of heme oxidation and/or extraction, has not been dramatically altered by hemophore binding. Since the quaternary structure of isolated HbCO is not known in our cryo-EM conditions, no further speculation is made on whether the R2-like structure observed in the IsdB:HbCO* complex should be considered an intermediate state on the path to that of IsdB:HbCO.

**Table 1.** Quaternary structure comparison between either IsdB:HbCO* (PDB ID 7PCQ) or IsdB:HbCO (PDB ID 7PCH) and reference Hb structures

Hb structure	PDB ID	α_1_β_1_ rmsd, Å		α_2_β_2_ rmsd, Å	
		IsdB:HbCO*	IsdB:HbCO	IsdB:HbCO*	IsdB:HbCO
IsdH bound	4XS0	2.077 [*1.284*]	1.841 [*1.086*]	9.471 [*9.407*]	7.893 [*7.750*]
T state	2DN2	1.748 [*1.080*]	1.485 [*0.862*]	7.198 [*7.099*]	5.628 [*5.468*]
IsdB bound	5VMM	1.811 [*1.187*]	1.533 [*0.950*]	3.766 [*3.574*]	4.390 [*4.258*]
R state	2DN1	1.579 [*0.878*]	1.190 [*0.619*]	3.108 [*2.850*]	1.833 [*1.524*]
R2 state	1BBB	1.325 [*0.737*]	1.696 [*1.027*]	2.680 [*2.246*]	4.292 [*4.087*]
R3 state	1YZI	1.961 [*1.282*]	1.804 [*1.107*]	5.078 [*4.931*]	4.514 [*4.281*]
		Rotation, °		Translation, Å	
IsdH bound	4XS0	22.45	17.85	5.62	5.13
T state	2DN2	17.26	13.15	2.82	2.05
IsdB bound	5VMM	9.30	12.33	0.64	0.80
R state	2DN1	6.69	3.45	1.29	0.74
R2 state	1BBB	5.06	9.59	0.42	0.95
R3 state	1YZI	14.33	13.05	1.27	1.26

rmsd values of all atom pairs are in regular type. rmsd values of C^α^ atom pairs are in italics.

### The IsdB:metHb Complex.

**Fig. 5.**
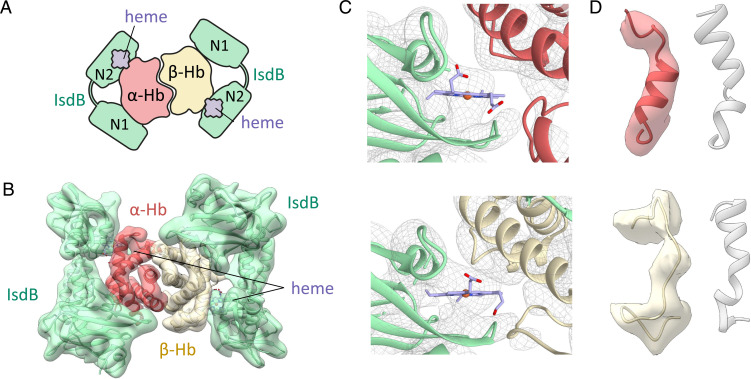
IsdB:metHb complex structure from cryo-EM. (*A*) Schematic representation of IsdB:metHb complex. (*B*) Atomic model refined through Flex-EM fitted in the 5.8 Å cryo-EM map. (*C*) Heme position in either α- (*Top*) or β-Hb (*Bottom*) subunits inside the cryo-EM map of the complex. (*D*) Comparison between F helices in either α- (*Top*) or β-Hb (*Bottom*) subunits within IsdB:metHb complex and native helices in isolated Hb structure (PDB ID 3P5Q) (40). The structure refined by cryo-EM inside the relative map density is colored similarly to *B*, while the native structure is in semitransparent gray.

The cryo-EM analysis of the IsdB:metHb complex yielded a map at estimated 5.8 Å resolution (*SI Appendix*, Fig. S4). While this did not allow precise location of side chains, the alignment of an ad hoc model with the map clearly indicates that the complex is formed by two IsdB molecules binding a Hb dimer (Fig. 5), an oligomeric state never structurally observed before. Different from the SEC-MALS experiments, here the higher protein concentration helped to stabilize the dimeric, rather than the monomeric, form of Hb. MetHb itself is expected to be mainly tetrameric at the concentration used for sample preparation, but, when complexed with IsdB, the presence of the Hb dimers might unveil a direct destabilizing effect of IsdB binding on Hb oligomerization state. The high protein concentration needed for structural studies in the crystal state might have thus hampered the identification of such a complex that, indeed, could be the physiologically relevant one (*S. aureus* feeds only on extracellular Hb). The interaction between IsdB and metHb was expected to lead to IsdB heme extraction, as supported by absorption spectroscopy (Fig. 1*G*) (4, 13); thus, the ad hoc model used for the interpretation of the cryo-EM map was prepared to have the cofactor bound to IsdB. Flexible fitting with Flex-EM improved the match of the atomic model and the volume density and allowed us to confidentially assign the heme position and to reveal structural rearrangement of F helices on both Hb chains (Fig. 5 *C* and *D*). The position of the heme in the ad hoc model was initially obtained from PDB ID 3RTL, the crystallographic structure of the heme-bound form of the isolated IsdB^N2^ domain (12), and fits well to the map after small shifts in refinement (Fig. 5*C*). The cryo-EM map thus describes a complex where the heme is bound to IsdB upon a completed extraction process. IsdB remains bound to Hb after heme extraction, and dissociation might require a slow structural rearrangement possibly including Pro173 *cis/trans* isomerization.

The extraction of heme from its binding pocket by IsdB requires that the F helix of Hb partially unfolds with breakage of the coordination bond between the proximal His and hemic iron. The initial alignment of the ad hoc model with the cryo-EM map revealed a great discrepancy in the region of the F helices. After flexible fitting, the model showed the partial unfolding of the F helix in the α-Hb chain, while the F helix in the β-Hb chain seems to be completely unfolded (Fig. 5*D*). This result confirms earlier findings (PDB ID 5VMM) (4) that heme transfer is associated with F-helix unfolding and additionally indicates that the unfolding persists on both Hb subunits after extraction and until IsdB dissociation. Assessment of local resolution in the IsdB:metHb complex density map shows that the IsdB^L^ and IsdB^N2^ domains of both hemophores have the lowest definition, possibly indicating the highest overall flexibility (*SI Appendix*, Fig. S4). IsdB^N2^ extracts the heme from Hb and, after this process is completed, this domain likely loses affinity for Hb and starts to dissociate. This evidence is in good agreement with molecular dynamics simulations (13), but also with the presence of a specific hinge region between IsdB^N1^ and IsdB^L^ domains that favors complex formation and heme extraction, keeping IsdB^N1^ stable and allowing the movement of IsdB^L^ and IsdB^N2^ domains (9, 15, 40).

## Discussion

**Fig. 6.**
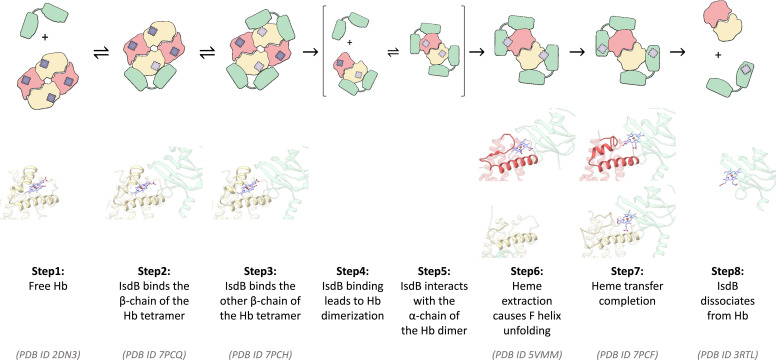
Hb binding and heme extraction by IsdB. The plausible sequence of events starting from IsdB binding to Hb and leading to heme extraction was built based on this work and on published data. A close-up of the heme-binding pocket and of the E and F helices of Hb, based on deposited 3D structures, is shown below the cartoons. The cryo-EM structure of IsdB:HbCO complex (PDB ID 7PCH) was used as a reference to align all the other PDB models. The alignment was based on Hb for steps 1 to 7, while IsdB was used for the alignment in step 8.

IsdB is a remarkable example of a receptor capable of catalyzing heme extraction from Hb by the orchestrated movement of the two IsdB^N1^ and IsdB^N2^ domains that, despite the high structural and sequence homology, play distinct roles along the heme removal pathway. The 3D structures that we describe here represent snapshots of two key structural states along the heme extraction process. The elusive preextraction complex (IsdB:HbCO complex) and the final state after heme transfer (IsdB:metHb complex) have been isolated, allowing visualization of the preparatory interactions for efficient heme extraction and the interactions that must eventually break to yield subsequent heme transfer to recipient proteins. In Fig. 6 the events along the heme extraction pathway are presented using structural data from this and published work.

IsdB can bind to tetrameric, ligated Hb (Fig. 6, step 1) in a stepwise manner (Fig. 6, steps 2 and 3), giving a final complex where Hb retains its quaternary structure and two IsdB molecules are bound to β-Hb subunits. An assembly with one IsdB molecule bound to one Hb tetramer was isolated by cryo-EM and is likely to represent an intermediate on the pathway to form a saturated complex. The assembly process is not known to be cooperative, but it may exhibit chelate-like cooperativity due to spatial colocalization of the IsdB on the cell surface (41). The accepted model of circulating Hb is an oxidized, dimeric form, as extracellular Hb is expected to be extremely diluted and prone to oxidation. However, *S. aureus* might release Hb from erythrocytes locally at the site of infection using hemolytic toxins (42). Thus, the Hb form used here to trap the initial complex between IsdB and Hb, i.e., HbCO, might mimic a physiological situation where IsdB can catch tetrameric oxyHb just released from red blood cells and promote its autoxidation (Fig. 1*F*).

The preference of IsdB for binding to either α-Hb or β-Hb has been largely debated and no conclusive results have been obtained so far, partly because methods used to trap intermediates invariably influence the chain preference of binding. In the only available structure of IsdB in complex with Hb, full-length IsdB chains bind to α-Hb; β-Hb subunits are engaged in a complex with just the IsdB^N1^ domain and the heme pocket of β-Hb is empty (4). This finding is strongly suggestive of an extraction event that occurred first at the β-Hb subunits followed by α-Hb subunits engagement. Our structures clearly indicate that the first binding event takes place on the β-Hb subunits and that α-Hb subunits are available for IsdB binding only within the dimeric Hb (Fig. 6, step 5). The molecular origin of this binding preference is likely a combination of elements. The type and number of interactions between IsdB and β-Hb identified in this work do not perfectly match with the interactions between IsdB and α-Hb identified by Bowden et al. (4) (*SI Appendix*, Fig. S5 *B* and *C*). Five interactions are lost in α-Hb [namely Lys8 (A5)-Glu190, Ser9 (A6)-Tyr165, Arg40 (C6)-Thr437, Glu43 (CD2)-Thr437, and Ser44 (CD3)-Thr365] and might account for the selectivity toward β-Hb. Indeed, Gell and coworkers reported that a single substitution of a key residue for the interaction of α-Hb with IsdH^N1^ is sufficient for completely abolishing binding (17). Furthermore, the arrangement of the two IsdB molecules on Hb following binding to α- and β-chains is completely different (*SI Appendix*, Fig. S5*A*); IsdB molecules bound to α-chains make extensive contacts that might have stabilized the complex during crystallization. However, the spatial proximity of the two IsdB molecules required for this arrangement might be less favorable in vivo, where IsdB is anchored to the cell wall. The preference of IsdB for binding β-Hb chains might exploit the natural propensity of β-Hb to release heme about 25-fold faster than α-Hb (14); indeed, some authors have speculated that heme release from β-subunits might take place without F-helix unfolding (21). However, our results and those by Murphy and coworkers (4) indicate that the F helix also unfolds on β-chains (Fig. 6, step 6) and remains unfolded in the final complex (Fig. 6, step 7). The F helix in the β-subunit is known to be more flexible than the corresponding region of the α-subunit and might favor the loosening of the coordination bond with the proximal histidine located on the same helix (16, 21, 43). Once the tight complex between IsdB and the β-Hb chains is formed, structural rearrangements, possibly including tetramer disruption, occur that enable subsequent binding of the hemophore to the α-Hb chains (Fig. 6, steps 4 and 5). IsdB binding to the β-chains of the tetramer could initiate the destabilization of the quaternary organization and the heme cavity rearrangement for the prosthetic group transfer to occur. It is well known that Hb tetramer disruption increases the hemic-iron autoxidation and the cofactor release rates (43–45) and, conversely, heme oxidation promotes tetramer disassembly (43, 46). Thus, the two processes are intertwined but our results suggest that specific binding to β-subunits primes heme extraction more by a direct effect on the heme microenvironment than on the stability of the tetramer. This result is apparently in contrast with earlier models where Hb dimerization is induced through the steric repulsion caused by the simultaneous binding of four hemophores on the Hb tetramer (23).

The structure of the IsdB:HbCO complex also reveals the presence of a *cis* peptide backbone conformation between Pro173 and Lys172 that has functional relevance in triggering Hb binding and/or complex dissociation once heme extraction is completed. In this conformation Lys172 establishes polar bonds with residues belonging to the Hb-binding motif, often indicated as loop 2. What we observed is an association between the folding state of loop 2 and the configuration of the Pro-Lys bond. In the structure presented here loop 2 folds in an α-helix and the bond is in *cis* configuration. Conversely, in the structures of the isolated IsdB^N1^ domain (PDB ID 2MOQ) (9) loop 2 is unstructured and the bond is in *trans* configuration. Establishing whether loop 2 folding or *cis/trans* isomerization takes place first will need further investigations. The IsdB^P173A^ variant shows impairment in both complex formation and dissociation after heme extraction, a finding that is strongly suggestive of a key role for Pro173 in the dynamics of complex formation and stability and might be required to facilitate or control the following steps in heme transfer to downstream acceptors.

The high-resolution structure reported here allowed the identification of the key contacts that stabilize the preextraction complex, some of which have never been reported before. This information might be exploited in the future for the identification of small molecules able to interfere with IsdB:Hb complex formation and thus cut off the iron supply necessary for *S. aureus* survival within the host. Moreover, the IsdB:HbCO complex reported here also provides an opportunity for further studies of the structure of hemoglobin in a liganded state at high resolution using single-particle methods. The high resolution, allowing to appreciate secondary-structure details, and the possibility offered by cryo-EM to determine structures in mixtures of conformations, might be the key for finally settling in the near future long-standing controversies on the heterogeneity of tertiary and quaternary states of Hb and the structural origin of high- and low-affinity conformations (47).

## Materials and Methods

Detailed materials and methods are included in *SI Appendix*.

### Cryo-EM Specimen Preparation.

IsdB was mixed with metHb in a 1:1 stoichiometric ratio (e.g., one IsdB molecule for each Hb chain) to reach a final concentration of 2 g/L, whereas the complex with HbCO was prepared at a final concentration of 8 g/L by mixing the hemophore with Hb in a 1:2 stoichiometric ratio. The different proportion between IsdB and Hb in the two complexes was chosen based on the results of SEC-MALS analysis.

Samples were prepared in 10 mM Hepes buffer (pH 7.3) to reduce the electron density of the background for cryo-EM data collection, and the zwitterionic detergent 3-[(3-cholamidopropyl)dimethylammonio]-2-hydroxy-1-propanesulfonate (CHAPSO) was added immediately before plunge freezing to a final concentration of 8 mM to overcome preferred orientation of the particles in the vitreous ice. A total of 3 µL of the complex solutions was applied onto glow-discharged 300-mesh R1.2/1.3 UltrAuFoil grids and then blotted for 3 s before rapidly cryocooling them in liquid ethane. For blotting and freezing the grids, a Vitrobot Mark IV (Thermo Fisher Scientific) device was used, and the sample chamber was kept at 4 °C and 99% humidity.

### Cryo-EM Data Collection.

For the high-resolution study, both IsdB:metHb and IsdB:HbCO complexes were imaged in a Titan Krios cryo-electron microscope (Thermo Fisher Scientific) at 300 kV, with a Gatan K3 direct electron detector working in superresolution mode at the calibrated magnification of ×130,000, yielding a pixel size of 0.326 Å. Micrographs were recorded by EPU software (Thermo Fisher Scientific) in movie mode, where each image was composed of 40 individual frames with an exposure time of 1.1 s and a dose rate of 15.3 electrons per second per Å^2^. The IsdB:metHb and IsdB:HbCO complex datasets resulted in a total of 3,050 and 2,852 movie stacks, with a defocus range of −1.0/−3.1 and −0.8/−2.8 μm, respectively.

### Image Processing.

The IsdB:HbCO complex was analyzed using WARP (48) to identify 460,000 particles, which were processed using cryoSPARC to classify two-dimensional (2D) and 3D classes and refine particle orientation parameters by imposing C2 symmetry (49). The 2D classification allowed us to separate two different states of IsdB:HbCO complex presenting a compositional heterogeneity. Within the first state, defined by 225,000 particles, the HbCO tetramer is bound by two hemophore molecules (IsdB:HbCO), while in the second state (90,000 particles) only one hemophore is binding the HbCO tetramer (IsdB:HbCO*). The maps were modified for interpretation using the auto_sharpen tools of the Phenix package (50).

The single-particle analysis of the IsdB:metHb complex was carried out using RELION (51). All micrographs were motion corrected using MOTIONCOR2 implemented in RELION and the contrast transfer function was estimated using Kai Zhang’s GCTF (52). Particles were found using the autopicking option in RELION, resulting in roughly 300,000 extracted particles, which were screened through multiple rounds of 2D and 3D classification to find the best set. The final refinement was run after the CFTRefine and Bayesian Polishing jobs in RELION with a final selection of roughly 100,000 particles.

Cryo-EM data processing yielded 2.9, 3.6, and 5.8 Å maps for the IsdB:HbCO, IsdB:HbCO*, and IsdB:metHb complexes, respectively. These resolutions for the final maps were estimated by the 0.143 criterion of the FSC curve.

### Model Building.

The atomic model of IsdB:HbCO complex was prepared by manually adjusting and refining in Coot (53) a starting model made using two different crystallographic structures, which describe IsdB:Hb complex (PDB ID 5VMM) (4) and native metHb (PDB ID 3P5Q) (54). The crystal structure of the protein complex is the only one published to date and presents a proteolyzed hemophore bound to the β-Hb chains and a native IsdB molecule bound to α-Hb chains. The proteolyzed portion of the hemophore was used to create a model with a native hemophore bound to β-Hb chains. The crystal structure of the IsdB:Hb complex also includes an Hb with missing atoms and unfolded portions; therefore, it has been replaced with the PDB coordinates of a native metHb (PDB ID 3P5Q) (54). Ultimately, the sequence of the hemophore in the model was modified to add the Strep-tag^®^ II, which was not present in the published structure. Water molecules were placed using Coot (53) and only those in agreement with the 2DN3 model (38) were maintained in the final structure.

The cryo-EM map density of the IsdB:metHb complex was not sufficiently detailed to accurately derive the position of the protein residues within the complex. Therefore, an ad hoc model where an Hb dimer was bound by two IsdB molecules (one for each Hb chain) was subjected to flexible fitting by use of Flex-EM (55) and then manually refined in Coot (53). The IsdB:metHb complex model was built starting from the PDB ID 5VMM structure (4). IsdB sequence, including the C-terminal Strep-tag^®^ II, was modeled on the IsdB template structure in 5VMM using the Swiss Model webserver. Since in 5VMM Hb secondary structure is severely altered, in our model we used native human hemoglobin structure (PDB ID 3P5Q) (54). Polar contacts were indicated if the distance between donor and acceptor was within 3.5 Å (hydrogen bonds) or 4.0 Å (ionic interactions). Nonpolar contacts were indicated between chains within 4.0 Å.

Atomic models were compared with published high-resolution structures using UCSF Chimera (56) and European Molecular Biology Laboratory-European Bioinformatics Institute (EMBL-EBI) PDBePISA (57). Images were prepared using the PyMOL Molecular Graphics System, Version 2.4.1 Schrödinger, LLC.

## Supplementary Material

Supplementary File

## Data Availability

The 3D cryoEM density maps generated in this study have been deposited in the Electron Microscopy Data Bank (EMDB) under accession codes EMD-13319, EMD-13320, and EMD-13325. The atomic coordinates have been deposited in the Protein Data Bank, with accession codes 7PCF, 7PCH, and 7PCQ. All study data are included in the article and/or *SI Appendix*.
