# Supplementary material for: Cryo-EM structures of staphylococcal IsdB bound to human hemoglobin reveal the process of heme extraction
Source: Proc Natl Acad Sci U S A. 2022 Mar 31;119(14):e2116708119. doi: 10.1073/pnas.2116708119 (PMC9168843; doi:10.1073/pnas.2116708119)
Supplement: Supplementary File [file pnas.2116708119.sapp.pdf]

PNAS

# Supplementary Information for

## Cryo-EM structures of staphylococcal IsdB bound to human hemoglobin reveal the process of heme extraction

Omar De Bei[a], Marialaura Marchetti[a,b,1], Luca Ronda[a,b,c,1], Eleonora Gianquinto[d,1], Loretta Lazzarato[d], Dimitri Y. Chirgadze[e], Steven W. Hardwick[e], Lee R. Cooper[e], Francesca Spyrakis[d], Ben F. Luisi[e], Barbara Campanini[a,f], Stefano Bettati[a,b,c]

[a]Interdepartmental Center Biopharmanet-TEC, University of Parma, Parma, 43124, Italy; [b]Department of Medicine and Surgery, University of Parma, Parma, 43126, Italy; [c]Institute of Biophysics, National Research Council, Pisa, 56124, Italy; [d]Department of Drug Science and Technology, University of Turin, Turin, 10125, Italy; [e]Department of Biochemistry, University of Cambridge, Tennis Court Road, Cambridge CB2 1GA, UK; [f]Department of Food and Drug, University of Parma, Parma, 43124, Italy.

[1]M.M., L.R. and E.G. contributed equally to this work

[2]To whom correspondence may be addressed. Email: barbara.campanini@unipr.it, bfl20@cam.ac.uk

**This PDF file includes:**

- Supplementary text
- Figures S1 to S6
- Tables S1 to S3
- SI References

## MATERIALS AND METHODS

**Protein expression and purification.** Expression and purification of Strep-tag® II-IsdB was carried out as previously described (1). Briefly, the sequence of the gene coding for the central and functional region of IsdB, residues 125-485, was retrieved from UniProt entry Q8NX66 (*S. aureus* MW2), optimized for expression in *E. coli* and subcloned in pASK-IBA3plus (IBA Lifesciences) vector, which led to the expression of IsdB with a C-terminal Strep-tag® II. *E. coli* cells transformed with pASK-IBA3plus::IsdB were grown at 37 °C until the mid-log phase ($OD_{600}$ = 0.5 – 0.6), and induced with 0.2 µg/mL anhydrotetracycline. Induction was performed for 20 hours at 20 °C. Cells were removed from the broth by centrifugation, resuspended in buffer W (100 mM Tris pH 8, 150 mM NaCl, 1 mM EDTA) in presence of 1 mg/mL lysozyme, and a protease inhibitor mix. Cell lysis was completed by sonication, and debris was removed by centrifugation. The purification was performed on a pre-equilibrated column, packed with Strep-Tactin®XT resin (IBA Lifesciences). Protein was eluted with 50 mM biotin in Buffer W. A final SEC step using a HiLoad 16/600 Superdex 75 prep grade column equilibrated in Buffer W was performed to remove high MW contaminants. The final protein preparation was over 95% pure, and the final yield of the purification was more than 100 mg/L of cell culture. The site-directed mutant P173A ($IsdB^{P173A}$) was prepared with standard mutagenesis techniques (2) and purified to homogeneity by the same method used for the wild type protein. IsdB concentration was calculated by using an extinction coefficient $\varepsilon_{280}$ = 47,790 $M^{-1}$ $cm^{-1}$ and MW = 43,320 g/mol. Since IsdB partially acquires heme from *E. coli* during overexpression, the amount of holo-IsdB in the preparation was estimated by calculating the heme concentration ($\varepsilon_{405}$ = 90,500 $M^{-1}$ $cm^{-1}$) (1) and resulted lower than 5% of the total amount of the protein.

Human Hb A was purified from outdated blood obtained from a local blood transfusion center. Only Hb from non-smoking donors was used, as described previously (3, 4). Briefly, red blood cells (RBC) were washed in saline solution and then lysed under hypotonic conditions (i.e., adding 7 volumes of Buffer Hb1 - 10 mM HEPES pH 6.9, 1 mM EDTA). The supernatant containing the oxygenated Hb was separated from cell debris by centrifugation, 1 h at 4 °C and 23,000 x g, and dialyzed against Buffer Hb1. The resulting solution was layered on a 100 X 5 cm CM-Sephadex C-50 column. Soluble RBC components were separated from Hb using a linear gradient from 0 to 80% of Buffer Hb2 (10 mM HEPES pH 8.6, 1 mM EDTA), while Hb was eluted using a linear gradient from 80 to 85% of Buffer Hb2. The purified protein was dialyzed against the storage buffer (10 mM HEPES pH 7.2, 1 mM EDTA), aliquoted, flash-frozen in liquid nitrogen and stored at −80 °C until further use. The concentration and the oxidation state of purified oxyHb was determined by UV-visible absorption spectroscopy by exploiting molar extinction coefficients for heme-characteristic absorption peaks (5). MetHb was obtained by oxidizing oxyHb in the presence of 5 mM potassium ferricyanide (Fluka). OxyHb was incubated for 10 minutes at room temperature in the presence of the oxidizing agent, the latter was removed on a Sephadex G-25 desalting column (GE Healthcare). Since metHb absorption spectrum depends on pH, the concentration of the protein was calculated spectroscopically by diluting the protein in buffer W, which is the buffer used for IsdB purification, where the extinction coefficient at 406 nm was calculated to be 130,000 $M^{-1}$ $cm^{-1}$ (Fig. S6). The entire analysis was performed in a sample chamber that consisted of a 2-mm optical path length cuvette fused to a 25-mL open-top threaded reservoir, where a screw cap with fittings for inlet and outlet gas lines can be mounted (6). The UV-Vis spectrum (240-750 nm) of oxyHb was recorded before proceeding with Hb deoxygenation. To obtain deoxygenated Hb (deoxyHb) a flow of helium was continuously passed over the Hb solution. During Hb deoxygenation, the sample chamber was thermostated

at 20 °C in a shaking bath. The completion of the reaction was confirmed by UV-Vis absorption spectroscopy. Finally, deoxyHb was oxidized by adding potassium ferricyanide to a final concentration of 5 mM. The final spectrum of metHb was acquired. OxyHb and deoxyHb UV-Vis spectra are independent of the buffer pH and were used to precisely calculate the Hb concentration inside the cuvette before the heme oxidation that led to metHb formation. HbCO was prepared by keeping oxyHb in a sealed glass chamber where a stream of pure carbon monoxide was passed over the solution. The reaction was carried out at 4 °C in the dark for 10 minutes by gently shaking the sample chamber to increase the diffusion of the gas into the solution and thus the ligand exchange. The completion of the reaction was confirmed by absorption spectroscopy by using tabulated molar extinction coefficients for heme-characteristic absorption peaks ($\varepsilon_{419}$ = 191,000 $M^{-1}$ $cm^{-1}$) (5).

**UV-Vis spectroscopy.** Spectroscopical characterization of heme extraction was carried out using a Cary 4000 spectrophotometer (Agilent). Heme extraction was already reported to cause a significant variation of visible absorption spectrum due to the different environment and coordination state of the cofactor upon transfer from Hb to IsdB (7). Absorption spectra (300-700 nm) of Hb and IsdB were recorded before and after mixing the two proteins at 20 °C. IsdB and metHb were mixed at 1:1 stoichiometric ratio (one IsdB molecule per each Hb subunit), while IsdB and HbCO were mixed at 1:2 ratio. The concentrations of the samples were 2 g/L and 8 g/L for the hemophore complex with oxidized and reduced form of Hb, respectively. The latter concentrations were used to be comparable with the solution used for grid preparation, and samples were diluted right before collecting spectra to avoid signal saturation.

**SEC and SEC-MALS.** Determination of molar masses of proteins was carried out by SEC-MALS. Single proteins and their complexes (100 µL at 1 g/L concentration) were loaded onto a Superdex™ 200 10/300 Increase GL SEC column (GE Healthcare) at 0.5 mL/min using an AKTA Purifier FPLC system (GE Healthcare). All protein solutions were centrifuged 30 minutes at 17,200 x g (4 °C) before loading, and protein complex solutions were allowed to react 15 minutes before centrifugation. Estimation of protein concentration was carried out using a NanoDrop® ND-1000 UV-Vis Spectrophotometer (Thermo Scientific). Along with SEC-MALS analysis, chromatograms were acquired and analyzed using UNICORN software (GE Healthcare) by recording the absorption signal at two different wavelengths, 280 nm for detecting the aromatic residues and 406 nm (metHb), 415 nm (oxyHb), or 419 nm (HbCO) to follow the elution of protein-bound heme at the wavelength of maximum absorption. Signals were exploited to extrapolate information about complex stoichiometry and heme extraction process. Absorption signals in the visible range were smoothed by applying Savitzky-Golay filters in MatLab®. To separate contributions of co-eluting proteins, the chromatograms were deconvoluted into single or multiple gaussians (Fityk software) (8). The solution eluted from the SEC column was analyzed with an online DAWN HELEOS II MALS detector (Wyatt Technology), followed by an Optilab T-rEX differential refractometer (Wyatt Technology) at 25 °C. The light scattering cell was illuminated by a 664 nm polarized laser source, and scattered light by the sample was detected by eight fixed angle detectors. Absolute and differential refractive indexes were calculated using a 658 nm LED light source. Data collection and analysis was performed using ASTRA 6 software (Wyatt Technology). The entire system was preconditioned with the experimental buffer and bovine serum albumin was used as a standard to confirm the system performance. On-line calculation of standard Zimm Plot (9), using a dn/dc value of 0.1850 mL/g, on eluted peaks from SEC column allowed the precise estimate of the molecular masses of separated samples.

**Hemoglobin autoxidation.** The oxyHb and HbCO autoxidation was evaluated by UV-Vis spectroscopy in PBS buffer (pH 7.4) at 37 °C using a Cary 4000 spectrophotometer (Agilent). Spectra (310-750 nm) in the absence or presence of IsdB (both Hb and IsdB at 7 μM concentration) were recorded every 30 minutes for up to 330 minutes. Data at all time-points were fitted to a linear combination of reference spectra corresponding to those of IsdB, oxyHb, HbCO, metHb, and IsdB:metHb complex recorded under the same experimental conditions. The fitting was performed using FMINCON in MatLab (10) to minimize the $\chi^2$ (eq 1):

$$(eq. 1) \qquad \chi^2 = \left(experimental\ spectrum - \sum par(i) * reference\ spectrum(i)\right)^2$$

The *par* in equation 1 are the coefficients of each *i* reference spectrum used. To evaluate the oxyHb and HbCO autoxidation, the coefficient of metHb spectrum expressed as a percentage is plotted as a function of time. Similarly, the Hb autoxidation in the presence of IsdB was evaluated considering the coefficient of IsdB:metHb complex, assuming that the hemophore rapidly extracts the heme as soon as Hb oxidizes. Unlike the experiment in which heme extraction was evaluated directly by mixing IsdB and metHb, autoxidation of Hb and cofactor transfer occur here.

**Enzyme-Linked ImmunoSorbent Assay (ELISA).** The affinity of IsdB and IsdB$^{P173A}$ for Hb was measured by an in-house developed method that exploits the Strep-tag® II to immobilize IsdB on Strep-Tactin®XT 96-well microplates (IBA Lifesciences). IsdB and IsdB$^{P173A}$ were incubated overnight in a microplate at the final concentration of 10 nM in 200 μL/well of a 2 mM EDTA, 140 mM NaCl, and 25 mM Tris/HCl, pH 7.6, solution (binding buffer). The following day, the binding buffer supplemented with 0.05% Tween-20 (washing buffer) was used to quickly wash the plate. OxyHb was then added at concentrations ranging from 50 pM to 100 nM in a final volume of 200 μL of binding buffer per well and was incubated for 1 h. Since IsdB binds oxyHb, but it is not able to extract the cofactor, this ligated form of Hb was used to evaluate complex formation without contribution from heme extraction. After plate washing with washing buffer, IsdB:Hb complexes were detected by 1 h incubation with horseradish peroxidase (HRP)-coupled anti-Hb polyclonal antibodies (Abcam) at 1:1000 dilution. After plate washing, bound oxyHb was quantified by measuring the catalytic conversion of 100 μL/well of a 3,3′,5,5′-tetramethylbenzidine (TMB, Acros Organics) solution, and the reaction was developed for 1 min. Subsequently, 100 μL/well of 1 N $H_2SO_4$ were applied to stop the reaction. All the steps before the plate reading were carried out at 4 °C. Absorption of the HRP reaction product was recorded at 450 nm with a microplate reader (Dynamica Halo LED 96), and background values from TMB solutions were subtracted from each reading. The non-specific binding of antibodies to hemophores, or non-specific signal caused by IsdB:Hb complexes were checked. For each Hb concentration, duplicate measurements were performed. The dissociation constant ($K_D$) was calculated by fitting data to a binding isotherm using SigmaPlot 12.5 Systat Software, Inc.

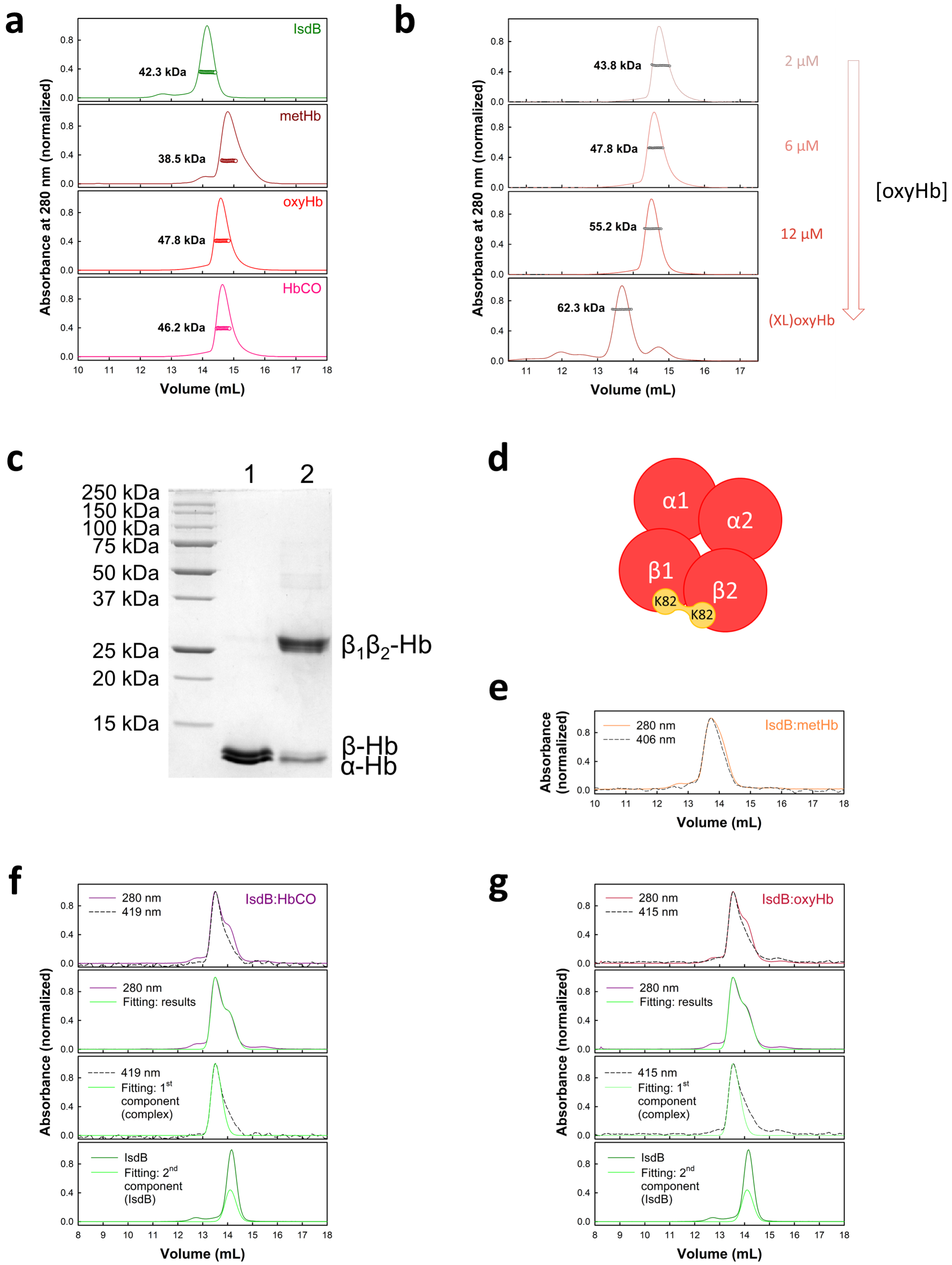


**Fig. S1. SEC-MALS analysis on isolated Hb or IsdB and IsdB:Hb complexes. a,** SEC-MALS analysis on isolated Hb and IsdB samples at 1 g/L concentration. Absorbance (lines) and weigh-average of MW (dots) are plotted versus the elution volume, showing constant molar-mass values over the entire peak width. **b,** SEC-MALS analysis of oxyHb samples at different concentration and of cross-linked (XL) oxyHb. **c,** SDS-PAGE of native oxyHb (lane 1) and (XL)

oxyHb (lane 2). **d,** Schematic representation of oxyHb crossed-linked on the β−subunits with bis (3,5-dibromosalycil) fumarate. **e,** Elution profile of IsdB:metHb complex measured at 280 nm (orange), overlaid with the elution profile measured at 406 nm (dashed line). **f,** From the top: (1) elution profile of IsdB:HbCO complex measured at 280 nm (purple) and at 419 nm (dashed line); (2) elution profile at 280 nm overlaid with the simulated elution profile obtained from Fityk fitting (green); (3) elution profile at 419 nm overlaid with the first component from the fitting (light green); (4) IsdB elution profile at 280 nm (dark green) overlaid with the second component from the fitting (light green). Legends in (3) and (4) indicate the proposed attribution of the reported curve fitting component. **g,** From the top: (1) elution profile of IsdB:oxyHb complex measured at 280 nm (dark red) and at 415 nm (dashed line); (2) elution profile at 280 nm overlaid with the simulated elution profile obtained from Fityk fitting (green); (3) elution profile at 415 nm overlaid with the first component from the fitting (light green); (4) IsdB elution profile at 280 nm (dark green) overlaid with the second component from the fitting (light green). Legends in (3) and (4) indicate the proposed attribution of the reported curve fitting component.

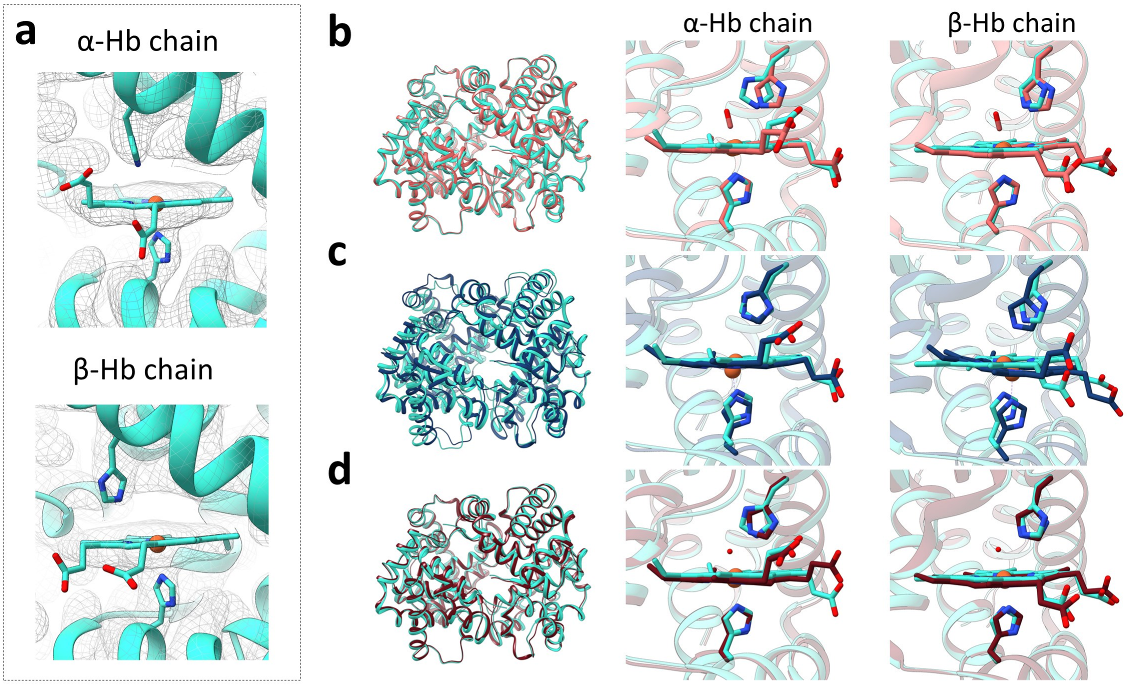


**Fig. S2. Hb ligation state in the cryo-EM map of IsdB:HbCO complex. a,** Atomic models and density maps of heme bound to α-Hb chain (top panel) and β-Hb chain (bottom panel) within the complex. **b, c, d,** Comparison between cryo-EM atomic model (cyan) and HbCO (PDB ID 2DN3 - red), deoxyHb (PDB ID 2DN2 – blue), or metHb (PDB ID 3P5Q – brown). From left to right: the quaternary structures, and the spatial configurations of proximal and distal histidines on α- and β-Hb chains.

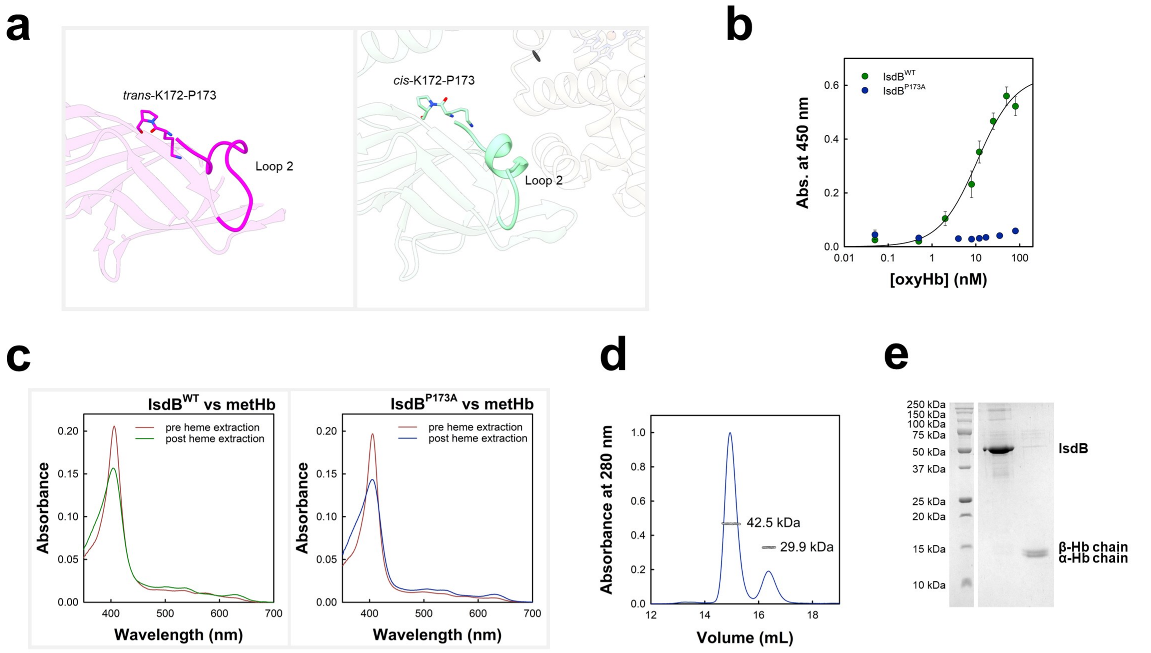


**Fig. S3. Characterization of the P173A variant of IsdB. a,** Comparison of isolated IsdB$^{N1}$ (PDB ID 2MOQ, left panel) with IsdB in complex with Hb (PDB ID 7PCQ, right panel). The structures are semi-transparent, while the K172-P173 peptide bond and the regions involved in folding upon binding (loop 2) are in color. **b,** Dependence of the HRP signal on Hb concentration for IsdB$^{WT}$ and P173A variant (IsdB$^{P173A}$). The dependence of IsdB$^{WT}$:oxyHb is fitted to a binding isotherm with $K_D$ = 11 nM (black line). **c,** Spectroscopic analysis of heme extraction from metHb by either IsdB$^{WT}$ (left panel) or IsdB$^{P173A}$ (right panel). Heme transfer from Hb to IsdB causes a decrease and a blue-shift of the Soret peak at 406 nm. **d,** SEC-MALS analysis of IsdB$^{P173A}$:metHb complex (1:1 stoichiometric ratio). **e,** SDS-PAGE of the peaks eluted in the SEC-MALS of panel d; lane 1: MW markers; lane 2: first peak (42.5 kDa); lane 3: second peak (29.9 kDa).

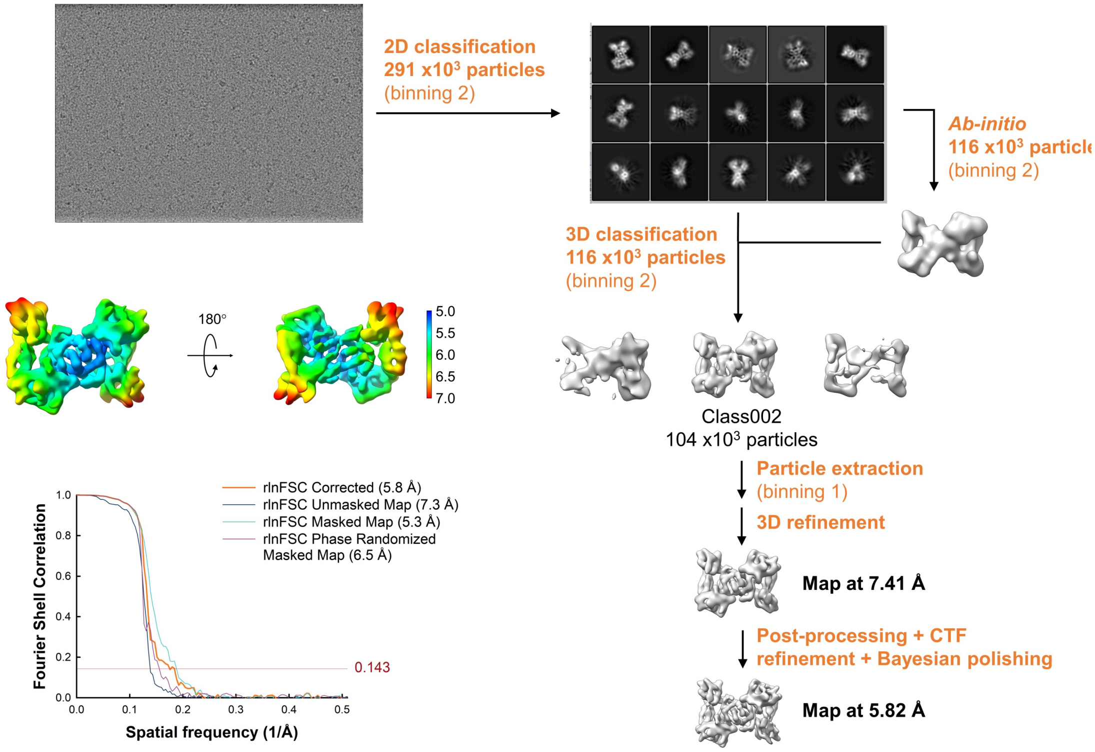


**Fig. S4. Single particle analysis of IsdB:metHb complex.** The top panel shows a representative micrograph and selected 2D class averages used to generate initial reference maps and refined maps (flow chart right). The middle right panel shows the local resolution cryo-EM density maps in two orientations for the IsdB:metHb (1:1) complex. The lower left panel shows the Fourier shell correlations for the model with different solvent masks, and the estimated resolution of the optimal reconstructions.

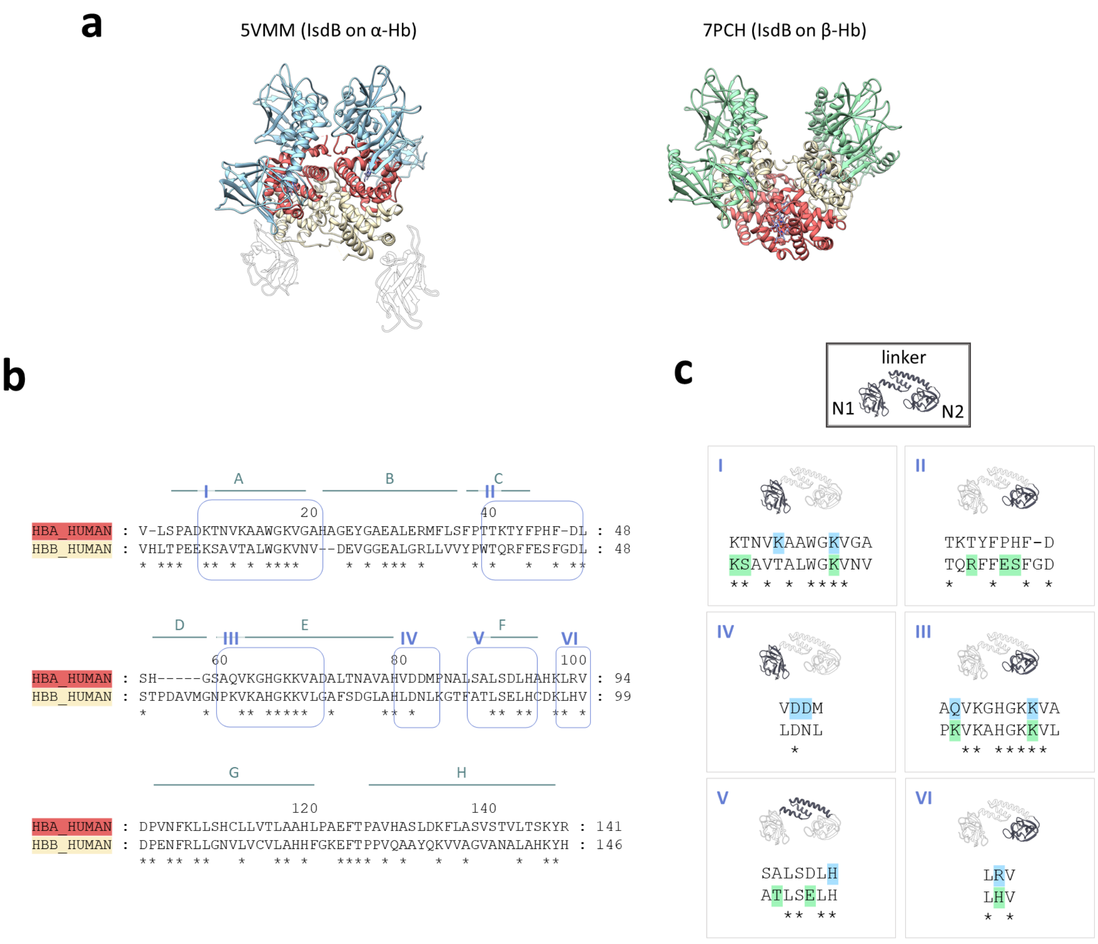


**Fig. S5. Binding of IsdB to either α-subunits or β-subunits of Hb. a,** Structures of IsdB in complex with Hb with IsdB binding to either α-Hb chains (PDB ID 5VMM, (7)) or β-Hb chains (PDB ID 7PCH, this work). **b,** Amino acid sequence alignment of α-Hb chain and β-Hb chain. Letters above the alignment indicate the α-helices of Hb. **c,** Close-up of the sequence alignment of human α-Hb chain and β-Hb chain showing residues involved in protein-protein interaction as retrieved from 5VMM (light blue) and 7PCH (green). Polar interactions were identified by analysis of the three-dimensional structures with the Pisa software followed by manual refinement. Two interactions with the F helix in region V are not present in 5VMM due to the unfolding of F helix that moves the residues away from the interface with IsdB$^{L}$.

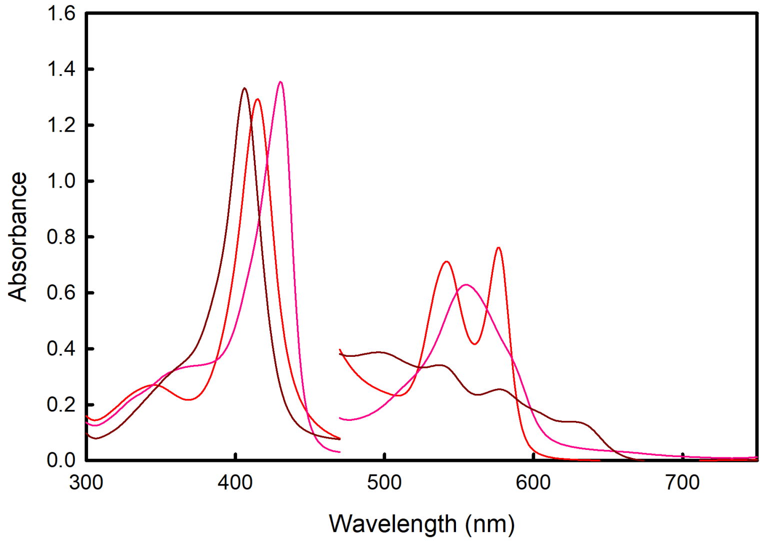


**Fig. S6. Spectra of metHb, oxyHb and deoxy Hb.** OxyHb (red), deoxyHb (magenta), and metHb (brown) spectra recorded in buffer W were used to estimate the metHb molar extinction coefficient. Absorption intensity from 465 nm to 750 nm was multiplied by a factor 5 to improve visualization of Hb Q bands.

**Table S1. Cryo-EM data collection, refinement, and validation statistics.**

| | IsdB:HbCO (PDB ID 7PCH) | IsdB:HbCO* (PDB ID 7PCQ) | IsdB:metHb (PDB ID 7PCF) |
|---|---|---|---|
| **Data collection and processing** | | | |
| Detector | Gatan K3 | Gatan K3 | Gatan K3 |
| Magnification | 130k | 130k | 130k |
| Energy filter slit width (eV) | 20 | 20 | 20 |
| Voltage (kV) | 300 | 300 | 300 |
| Flux on detector ($e^-$/pix/sec) | 15.3 | 15.3 | 15.3 |
| Electron exposure on sample ($e^-/Å^2$) | 39.59 | 39.59 | 39.59 |
| Target defocus range (μm) | 0.8-2.8 | 0.8-2.8 | 1.0-3.1 |
| Calibrated pixel size (Å) | 0.652 | 0.652 | 0.652 |
| Software | WARP/ cryoSPARC | WARP/ cryoSPARC | Relion |
| Symmetry imposed | C2 | C1 | C1 |
| Extraction box size (pixels) | 360 | 360 | 250 |
| Number of micrographs | 2852 | 2852 | 3050 |
| Initial particle images (no.) | 622246 | 622246 | 521822 |
| Final particle images (no.) | 224937 | 89975 | 103653 |
| Map resolution, $FSC_{0.143}$ (Å) | 2.9 | 3.6 | 5.8 |
| **Refinement** | | | |
| Software | Coot/Phenix | Coot/Phenix | Coot/Phenix |
| Initial model used | Ad hoc model based on 5VMM, 3P5Q, and 2DN3 | Ad hoc model based on 5VMM, 3P5Q, and 2DN3 | Ad hoc model based on 5VMM, and 3P5Q |
| Model resolution, $FSC_{0.143}$ (Å) | 3.0 | 3.6 | 4.6 |
| **Model composition** | | | |
| Protein residues | 1254 | 911 | 968 |
| Ligands | HEM: 4 | HEM: 4 | HEM: 2 |
| Molar mass (kDa) | 151.2 | 107.9 | 118.9 |
| **R.m.s. deviation** | | | |
| Bond lengths (Å) | 0.013 | 0.013 | 0.014 |
| Bond angles (°) | 1.550 | 1.544 | 1.600 |

| **Validation** | | | |
|---|---|---|---|
| MolProbity score | 1.02 | 1.00 | 0.72 |
| Clashscore | 0.97 | 1.30 | 0.28 |
| Rotamers outliers (%) | 0.55 | 0.78 | 0.00 |
| **Ramachandran plot** | | | |
| Favored (%) | 96.46 | 97.23 | 97.40 |
| Allowed (%) | 3.54 | 2.77 | 2.60 |
| Disallowed (%) | 0.00 | 0.00 | 0.00 |

**Table S2. Principal polar interactions between IsdB and Hb, grouped according to the position in Fig. 3.**

| β-Hb (chain B) | IsdB (chain E) | Distance (Å) | Zone |
|---|---|---|---|
| Lys 8 (A5)/NZ/1219 | Gln 190/OE1/2906 | 3.46 | |
| Ser 9 (A6)/OG/1225 | Tyr 165/OH/2702 | 3.44 | **1** |
| Lys 17 (A14)/NZ/1284 | Glu 247/OE1/3377 | 3.25 | |
| Thr 87 (F3)/OG1/1839 | Tyr 293/OH/3780 | 3.15 | |
| Thr 87 (F3)/OG1/1839 | Lys 297/NZ/3814 | 3.47 | |
| Glu 90 (F6)/OE2/1863 | Tyr 293/OH/3780 | 3.33 | **2** |
| His 97 (FG4)/NE2/1922 | Asp 439/OD2/4980 | 3.57 | |
| His 97 (FG4)/NE2/1922 | Asp 439/O/4976 | 3.71 | |
| Arg 40 (C6)/NH2/1465 | Thr 437/O/4961 | 2.75 | |
| Glu 43 (CD2)/OE1/1495 | Thr 437/OG1/4963 | 3.48 | |
| Hem 147/O1D/2353 | Tyr 444/OH/5025 | 2.72 | **3** |
| Hem 147/O1D/2353 | Tyr 440/OH/4992 | 2.39 | |
| Hem 147/O2D/2355 | Tyr 444/OH/5025 | 2.79 | |
| Ser 44 (CD3)/O/1500 | Thr365/OG1/4369 | 3.38 | |
| Lys 59 (E3)/NZ/1609 | Ser361/O/4337 | 3.2 | |
| Lys 59 (E3)/NZ/1609 | Thr365/OG1/4369 | 3.07 | **4** |
| Lys 66 (E10)/NZ/1680 | Glu354/OE2/4286 | 2.99 | |

**Table S3. Principal apolar interactions between IsdB and Hb, grouped according to the position in Fig. 3.**

| β-Hb (chain B) | IsdB (chain E) | Distance (Å) | Zone |
|---|---|---|---|
| Ala 10 (A7)/CA | Phe 242/CD1 | 3.97 | 1 |
| Ala 10 (A7)/CA | Phe 242/CE1 | 3.87 | |
| Ala 10 (A7)/CB | Phe 242/CE1 | 3.98 | |
| Ala 13 (A10)/CA | Tyr 165/CD1 | 3.8 | |
| Ala 13 (A10)/CB | Phe 242/CD1 | 3.66 | |
| Leu 14 (A11)/CD1 | Phe 242/CZ | 3.77 | |
| Trp 15 (A12)/CB | Phe 164/CD2 | 3.85 | |
| Trp 15 (A12)/CB | Phe 164/CE2 | 3.86 | |
| Trp 15 (A12)/CD1 | Phe 164/CG | 3.99 | |
| Trp 15 (A12)/CD1 | Phe 164/CD2 | 3.91 | |
| Trp 15 (A12)/CD1 | Phe 164/CE2 | 3.99 | |
| Leu 75 (E19)/CB | Phe 164/CZ | 3.56 | |
| Ala 76 (E20)/CB | Tyr 167/CE2 | 3.72 | |
| Phe 41 (C7)/CD1 | Ile 438/CG2 | 3.90 | 3 |
| Leu 96 (FG3)/CD2 | Tyr 440/CD1 | 3.92 | |

1. E. Gianquinto, *et al.*, Interaction of human hemoglobin and semi-hemoglobins with the Staphylococcus aureus hemophore IsdB: a kinetic and mechanistic insight. *Sci. Rep.* **9**, 18629 (2019).
2. H. Liu, J. H. Naismith, An efficient one-step site-directed deletion, insertion, single and multiple-site plasmid mutagenesis protocol. *BMC Biotechnol.* **8**, 91 (2008).
3. J. E. Fuhr, P. Medici, An in vitro effect of thyroid hormone upon bone marrow synthesis of hemoglobin. *FEBS Lett.* **11**, 20–22 (1970).
4. C. Viappiani, *et al.*, Experimental basis for a new allosteric model for multisubunit proteins. *Proc. Natl. Acad. Sci.* **111**, 12758–12763 (2014).
5. E. Antonini, M. Brunori, *Hemoglobin and Myoglobin in Their Reactions with Ligands* (North-Holland Pub. Co, 1971).
6. L. Ronda, S. Bruno, S. Faggiano, S. Bettati, A. Mozzarelli, “Chapter 16 - Oxygen Binding to Heme Proteins in Solution, Encapsulated in Silica Gels, and in the Crystalline State” in *Globins and Other Nitric Oxide-Reactive Proteins, Part B*, Methods in Enzymology., R. K. Poole, Ed. (Academic Press, 2008), pp. 311–328.
7. C. F. M. Bowden, *et al.*, Structure–function analyses reveal key features in Staphylococcus aureus IsdB-associated unfolding of the heme-binding pocket of human hemoglobin. *J. Biol. Chem.* **293**, 177–190 (2018).
8. M. Wojdyr, Fityk : a general-purpose peak fitting program. *J. Appl. Crystallogr.* **43**, 1126–1128 (2010).
9. S. Girod, P. Baldet-Dupy, H. Maillols, J.-M. Devoisselle, On-line direct determination of the second virial coefficient of a natural polysaccharide using size-exclusion chromatography and multi-angle laser light scattering. *J. Chromatogr. A* **943**, 147–152 (2002).
10. *MATLAB and Optimization Toolbox Release 2021a* (The MathWorks, Inc.).
